# Genome-wide association studies reveal differences in genetic susceptibility between single events vs. recurrent events of atrial fibrillation and myocardial infarction: the HUNT study

**DOI:** 10.3389/fcvm.2024.1372107

**Published:** 2024-04-25

**Authors:** Martina Hall, Anne Heidi Skogholt, Ida Surakka, Haavard Dalen, Eivind Almaas

**Affiliations:** ^1^ Department of Biotechnology and Food Science, Norwegian University of Science and Technology, Trondheim, Norway; ^2^ K.G. Jebsen Center for Genetic Epidemiology, Norwegian University of Science and Technology, Trondheim, Norway; ^3^ Department of Internal Medicine, University of Michigan, Ann Arbor, MI, United States; ^4^ Clinic of Cardiology, St. Olavs University Hospital, Trondheim, Norway; ^5^ Department of Circulation and Medical Imaging, Norwegian University of Science and Technology, Trondheim, Norway; ^6^ Department of Medicine, Levanger Hospital, Nord-Trøndelag Hospital Trust, Levanger, Norway

**Keywords:** GWAS meta-analysis, recurrent events, atrial fibrillation, myocardial infarction, networks, PheWAS, HUNT

## Abstract

Genetic research into atrial fibrillation (AF) and myocardial infarction (MI) has predominantly focused on comparing afflicted individuals with their healthy counterparts. However, this approach lacks granularity, thus overlooking subtleties within patient populations. In this study, we explore the distinction between AF and MI patients who experience only a single disease event and those experiencing recurrent events. Integrating hospital records, questionnaire data, clinical measurements, and genetic data from more than 500,000 HUNT and United Kingdom Biobank participants, we compare both clinical and genetic characteristics between the two groups using genome-wide association studies (GWAS) meta-analyses, phenome-wide association studies (PheWAS) analyses, and gene co-expression networks. We found that the two groups of patients differ in both clinical characteristics and genetic risks. More specifically, recurrent AF patients are significantly younger and have better baseline health, in terms of reduced cholesterol and blood pressure, than single AF patients. Also, the results of the GWAS meta-analysis indicate that recurrent AF patients seem to be at greater genetic risk for recurrent events. The PheWAS and gene co-expression network analyses highlight differences in the functions associated with the sets of single nucleotide polymorphisms (SNPs) and genes for the two groups. However, for MI patients, we found that those experiencing single events are significantly younger and have better baseline health than those with recurrent MI, yet they exhibit higher genetic risk. The GWAS meta-analysis mostly identifies genetic regions uniquely associated with single MI, and the PheWAS analysis and gene co-expression networks support the genetic differences between the single MI and recurrent MI groups. In conclusion, this work has identified novel genetic regions uniquely associated with single MI and related PheWAS analyses, as well as gene co-expression networks that support the genetic differences between the patient subgroups of single and recurrent occurrence for both MI and AF.

## 1 Introduction

Myocardial infarction (MI) and atrial fibrillation (AF) are two prevalent cardiovascular diseases. AF, in particular, is a well-established risk factor for several other cardiovascular conditions. The severity and mortality risk associated with AF increase significantly upon relapse. To manage and prevent new AF events, a range of medical and interventional therapies are available. These treatments aim to either normalize the rhythm or stabilize the heart rate. Among these approaches, AF ablation has emerged as a leading clinical treatment. However, its success rate varies, and approximately 20%–40% of patients may require additional treatment (1). Similarly, MI is a severe heart diagnosis associated with high mortality rates. Upon survival, the heart is most likely weakened, making the patient vulnerable to other diseases. In fact, 33% of patients experiencing MI die within a year (all causes of death) (2). However, some patients experience only a single event of MI and lead a normal and healthy life afterward. Most MI patients undergo cardiac catheterization and percutaneous coronary interventions in the acute phase, while medical therapies targeting clotting of blood and lipids, as well as lifestyle interventions, are provided to reduce the chance of recurrent events. Still, a significant proportion of MI patients suffer from relapse.

Many studies have been conducted to identify genetic variants that likely affect the risk of AF (3, 4) and MI (5, 6). While multiple variants have been identified and later replicated in other studies, these variants were identified when comparing all included cases of AF or MI with healthy controls. Some studies have been conducted to understand the genetics of patients experiencing recurrent AF (1, 7–10) and MI events (11–13). These are, however, mostly focused on either the patients’ response after treatment or the genetic effects on recurrence from known AF/MI variants or genes. Little effort has been made regarding the comparison of genetics between patients experiencing a single event vs. patients experiencing recurrent events.

To date, no comprehensive genome-wide association studies (GWAS) analysis has directly compared the genetic profiles of patients with recurrent events to those experiencing single events in either AF or MI. In this study, we explore whether statistically significant genetic differences exist between patients who encounter recurrent events (defined as two or more occurrences) of AF or MI and patients who only experience a single event. Notably, we do not differentiate cases based on the specific treatment received after the initial AF or MI event. By adopting this approach, we aim for a broad comparison to uncover potential genetic distinctions between patients with single events and those with recurrent events.

## 2 Methods

### 2.1 Cohorts

#### 2.1.1 The HUNT study

The Trøndelag Health study (HUNT) is a health-related population-based longitudinal study based on four rounds of data collection: HUNT1 (1984–1986), HUNT2 (1995–1997), HUNT3 (2006–2008), and HUNT4 (2017–2019). With a unique database covering clinical measurements, questionnaire data, and biological samples from roughly 230,000 inhabitants of the Trøndelag county from 1984 onward, it is one of the largest health study ever performed (14). A great benefit of the HUNT study is the connection to other health-related registries by use of the Norwegian unique personal identification number. These health registries include hospital and general practitioner registries, cancer registries, cause of death registries, and the prescription database.

In the current study, genotype data for 69,621 participants from HUNT2 and HUNT3 were used, and these were linked to questionnaire data and clinical measurements from HUNT1, HUNT2, and HUNT3, regional hospital records, the Nord-Trøndelag Hospital Trust (HNT), and the Norwegian Cause of Death Registry (COD). The HNT registry contains all ICD9 and ICD10 codes for hospital visits of these HUNT participants from August 1987 to April 2017. The COD registry spans the same period, with registered ICD9 and ICD10 codes for the primary and secondary causes of death.

#### 2.1.2 The United Kingdom Biobank

The United Kingdom Biobank (UKBB) is a health-related population-based study consisting of approximately 500,000 middle-aged UK inhabitants. Sampling of the participants took place from 2006 to 2010, when questionnaires, clinical measurements, and biological samples were collected. Similar to the HUNT study, it is also linked to electronic health records that contain information about the participants’ hospital, general practitioner, and death records with ICD9 and ICD10 diagnose codes (15). Genotyped data are available for more than 480,000 of the participants, and in the current study, we use these data together with relevant questionnaire data, clinical measurements, and hospital and death records for the genotyped participants (of European ancestry). The hospital records span from December 1992 to September 2021. The death registry spans from 2006 until September 2021.

### 2.2 Genotyping and imputation

Genotyping and imputation of the HUNT and UKBB participants have been described elsewhere (16, 17). Briefly, genotyping was performed using one of three Illumina HumanCoreExome arrays: 12 v.1.0, 12 v.1.1 with custom content (UM HUNT Biobank v1.0) according to standard protocols for the HUNT participants, and standard protocols for Affymetrix Applied Biosystems UK BiLEVE Axiom or Applied Biosystems UK Biobank Axiom array for the UKBB participants. Standard quality control was performed for the HUNT genotyping, as well as a UKBB-specific quality control for the UKBB genotyping. Imputation in HUNT was performed using 2,202 whole-genome sequenced samples from HUNT together with the Haplotype Reference Consortium (HRC) reference panel (18, 19), resulting in 25 million genetic markers. For UKBB, the HRC and UK10K+1000 Genomes reference panel were used, resulting in 90 million variants.

### 2.3 Definitions of traits and outcomes

Hospital records of HUNT and UKBB participants were used to determine cases of MI and AF as well as the number of events for each participant. An MI event is defined as the patient having a registered diagnosis of ICD10:I21-I24 or ICD9:410. An AF event is defined as a diagnosis of ICD10:I48 or ICD9:427.3.

In both UKBB and HUNT records, each diagnosis is registered as a main or bi-diagnosis (denoted as first and second diagnosis in UKBB), and we take both of them into consideration when determining the number of events for each participant. Limiting our analysis to only the main diagnosis while excluding bi-diagnoses introduces potential errors and significantly reduces the available data. Bi-diagnoses can be interpreted in various ways. For instance, consider a scenario where a patient is admitted to the hospital with a primary diagnosis, and additional diagnoses are identified and documented during that initial visit. Despite being new diagnoses, these are categorized as bi-diagnoses. Had they been the sole disease and reason for the hospital visit on that day, they might have been recorded as the main diagnoses. Alternatively, a physician might infer from historical records that certain other conditions (such as AF or MI) are relevant to the primary diagnosis and include them as bi-diagnoses, even if they are not recent events. Given the diverse reporting practices related to bi-diagnoses, we employ selective filtering to distinguish single events from recurrent events of MI and AF.

A first event is defined as the initial visit during which a specific diagnosis appears in the medical records, either as a primary (main) or as a secondary (bi-diagnosis) diagnosis. Subsequently, a second event is established if there exists a time gap of more than 1 month between the initial event and any subsequent occurrences. The second event must meet one of the following criteria: (i) it is recorded as a main diagnosis or (ii) it is a bi-diagnosis and the sole diagnosis documented on that particular day (indicating a genuinely new event reported at that time). Subsequent events are similarly categorized as second events, with a minimum interval of 1 month from the previously defined event. This event definition ensures the selection of new occurrences, except in cases where only main diagnoses are exclusively considered.

In our investigation of comparing patients with recurrent events to those who remain relapse-free, it is crucial to address the potential misclassification of patients with only a single recorded event. Specifically, we need to ensure that such patients are not erroneously categorized due to premature mortality before experiencing subsequent events. Analyzing the HUNT dataset, we observe that approximately 80% of secondary events occur within a 5-year window for AF and a 7-year window for MI. To mitigate this potential bias, we apply the following filtering criteria: First, we exclude single-event participants who have passed away either due to the phenotype itself or within the specified time frames after the initial AF or MI event. These time frames align with the observed secondary event patterns in the HUNT and UKBB datasets. Second, we remove participants registered with only a single AF or MI event if it occurred less than 5 (AF) or 7 (MI) years before the censoring dates (*6 April 2017* for HUNT and *12 November 2021* for UKBB). The three trait groups are denoted as single AF/MI: participants that experience only one event of AF/MI, and recurrent AF/MI: participants that experience more than one event of AF/MI, while satisfying the conditions specified above.

Baseline and clinical characteristics, as well as information about other relevant diseases identified with the participants, were taken from the HUNT and UKBB hospital records, questionnaires, and clinical measurements. Participants were defined to have diabetes and/or hypertension if they have ever been registered with the ICD codes ICD10:E10-E14 or ICD9:250 for diabetes and/or ICD10:I10-I15 (excluding I11.0), and ICD9:401-405 for hypertension. The smoking variable was derived from the HUNT questionnaire response to the question: “Have you ever smoked?” (with options for “Yes” or “No”). For each patient, we utilized the most recent HUNT participation data available prior to the disease event. The corresponding variable in UKBB was “Ever smoked (Yes/No),” which was constructed upon sampling. To assess statistical differences in characteristics (age, diabetes, hypertension, BMI, smoking, cholesterol, systolic, and diastolic blood pressure) between groups with single vs. recurrent events, we employed the Student’s t-test for continuous variables and Fishers’ exact test for binary variables. Test statistics with Bonferroni-adjusted p-values ($p<0.05/8=6.25\times 10^{-3}$) were considered significant findings.

### 2.4 GWAS meta-analysis

To identify genetic factors associated with single or recurrent events of AF and MI, we conducted three GWAS analyses for each trait in both cohorts separately: (i) patients with single events vs. healthy controls, (ii) patients with recurrent events vs. healthy controls, and (iii) patients with recurrent events vs. patients with single events. As a control, we also conducted a GWAS analysis in each cohort with all cases of each disease against healthy controls. Healthy controls were defined as participants with no registered events of AF and MI. Variants with minor allele count (MAC) <3 and an imputation score <0.3 were excluded from all GWAS results. Participants with non-European recent ancestry were excluded from the analyses in UKBB (note that all genotyped HUNT participants are of European ancestry). Association analyses were performed with SAIGE, using a generalized linear mixed model adjusted for relatedness and unbalanced case–control ratios (20). Birth year, gender, batch/chip, and the first four principal components were added as covariates in the models. Here, birth year is chosen instead of age at the time the event was recorded to facilitate the building of phenotypes based on a heterogenic set of data sources collected at different time points using multiple diagnostic codes. Genomic variants with minor allele frequency (MAF) >1% in one or both studies were included in the meta-analysis.

From the eight GWAS analyses (three for AF, three for MI, and one control for each disease) performed for both the HUNT population and the UKBB population, we performed eight fixed-effect inverse variance weighted (IVW) meta-analyses using METAL (21). In METAL, each variant is assigned a new effect size as the sum of each study’s effect size weighted by the corresponding study variance. The p-values in the meta-analysis are calculated based on the Z statistic given by the new effect sizes and standard errors. Variants reaching genome-wide significance (p-values $<5\times 10^{-8}$) from the Z statistic were considered significant findings. Annotations of significant single nucleotide polymorphisms (SNPs), identification of nearest genes, and a search for nearby SNPs associated with relevant traits were performed with the FUMA platform and the GWAS catalog (22, 23). Variants were considered to be in the same genetic region if they were less than 500 kb apart, and genetic regions denoted as shared for both the single and the recurrent events meta-analysis were either consisting of the same SNPs or SNPs within the same genetic region. Observed scale genetic heritability of the traits were found using the LD Score Regression software (24), with precomputed LD Scores for Europeans from the 1000 Genomes reference panel (25) and summary statistics from the meta-analysis.

### 2.5 Phenome-wide association studies

Phenome-wide association studies (PheWAS) were performed on all SNPs from the meta-analyses reaching genome-wide significance. From the comprehensive Pan UKBB resource (26), we collected results from GWAS conducted on 1,326 phenocodes, and we identified the effect of each of our SNPs of interest on each phenocode. All GWAS results from the Pan UKBB are based on UKBB participants, and we selected results for European ancestry exclusively. For each set of SNPs (identified in common or specifically for either single or recurrent AF/MI), phenotypes with a p-value $<0.05/(1326\times n_{\mathrm{set}})$, where $n_{\mathrm{set}}$ is the number of SNPs in the set, were considered significant associations. For simplicity, only the SNP with the lowest p-value for each phenotype was selected from each set of SNPs.

### 2.6 Gene function and network analyses

The sets of nearest genes to the SNPs identified through the GWAS analyses as common or unique to either recurrent or single AF/MI events (in total six sets) were analyzed for tissue specificity (differentially expressed gene sets in each tissue). We employed both FUMA (22) and gene ontology enrichment using Fisher’s exact over-representation test in PANTHER (protein annotation through evolutionary relationship) (27). Here, biological processes with a false discovery rate (FDR) adjusted for multiple testing <0.05 were considered functionally enriched for the gene set. To further investigate the processes connected to these genes, we performed gene co-expression network analysis (28–30), where the hypothesis is that highly correlated genes have a regulatory relationship or similar response in a condition (31). Using the identified gene sets as target genes in an egocentric gene co-expression network analysis, we generated a network from the shared neighborhoods among the closest neighbor genes of each target gene in the gene set, and we inspected the gene functions in the network.

Creating these egocentric networks involves several steps. First, using gene expression data from GTEx v.8 (32) (https://www.gtexportal.org) gene co-expression networks for seven tissue sub-types from the heart, muscle, skeletal, artery, and kidney (GTEx_Analysis_v8_eQTL_expression_matrices.tar: *Heart Atrial Appendage, Heart Left Ventricle, Muscle Skeletal, Artery Aorta, Artery Coronary, Artery Tibial*, and *Kidney Cortex*) were created. Since co-expression patterns may vary in different tissues (31), a separate network was created for each tissue. Following the WGCNA approach (33), the link weight (strength of co-expression) between each pair of genes (i and j) were defined by the weighted topological overlap (wTO) in Equation 1:

(1) $${\text{wTO}}_{ij}=\frac{A_{ij}+\sum_{k\neq i,j}A_{ik}A_{kj}}{\min(\sum_{u}A_{iu},\sum_{u}A_{\;ju})+1-A_{ij}},$$

where $A_{ij}=|\text{cor}(i,j){|}^{6}$ is the absolute Pearson correlation of the gene expressions raised to a power 6 to emphasize the strongest correlations. The resulting gene co-expression network is then an all-to-all network where pairs of genes with high wTO-link weights represent strong connections between the genes and their topological neighborhood. Only the 15% strongest links from each tissue were included in the following analysis (still leaving about 30 million links) to avoid the inclusion of genes based on weak (and likely spurious) connections.

Next, for each of the seven tissues, egocentric networks for each target gene were extracted from the co-expression networks. The egocentric networks were filtered to include only the 25 genes with the strongest wTO-link weights with each target gene. By merging and further reducing the complexity of the networks, the 25 strongest linked genes to each target gene across all tissues were selected in the final network. Here, we weighted the link strengths using ${\text{wTO}}_{ij}^{\mathrm{weighted}}=\sqrt{\sum_{w=1}^{W}\;{\text{wTO}}_{ij,w}^{2}}$, where W is the number of tissues in which the linked gene is among the 25 strongest linked genes to the target gene and ${\text{wTO}}_{ij,w}$ is the corresponding wTO-link weight in tissue w.

The final six sets of egocentric networks for target genes identified as common or as unique for the single or recurrent AF/MI events were analyzed with the *igraph* R-package (34, 35). Shared neighboring genes were defined as genes linked to two or more of the target genes. The set of shared neighborhood genes for each network was plotted separately with Cytoscape (v. 3.8.1) (36) and gene ontology enrichment of these gene sets were obtained through the PANTHER over-representation test (27).

## 3 Results

### 3.1 Characteristics of trait groups

Among the genotyped participants with European ancestry included in this study, there are 7,127 and 29,330 hospital patients registered with AF in HUNT and UKBB, respectively. Employing the filtering approach described in the Methods section, we identified 1,425 HUNT and 9,561 UKBB patients with single AF events and 2,267 HUNT and 7,267 UKBB patients with recurrent AF events. Correspondingly, 5,805 HUNT and 14,592 UKBB participants are registered with MI events. Of these, 1,651 HUNT and 6,584 UKBB patients are identified with single MI events and 1,615 HUNT and 1,615 UKBB patients are identified with recurrent MI events.

Baseline and clinical characteristics of these patients are presented in Table 1. In the HUNT study, a comparison between the two AF groups reveals a discernible pattern. The group experiencing a single AF episode tends to be older (adjusted p-value $2\times 10^{-10}$) and displays elevated levels of cholesterol and systolic blood pressure (adjusted p-values $6.1\times 10^{-6}$ and $5\times 10^{-4}$, respectively). Similarly, an examination of the AF groups within the UKBB reinforces this trend, with the single AF event group exhibiting higher age (adjusted p-value $<10^{-16}$), along with marginally higher levels of BMI and systolic blood pressure compared to the recurrent AF group.

**Table 1:** Characteristics of sample groups of single and recurrent events of AF and MI in the HUNT and UKBB population.

	AF HUNT		AF UKBB	
	Single events	Recurrent events	Single events	Recurrent events
	n=1,435	n=2,267	n=9,561	n=7,267
Male (%)	58	62	65	66
Birth year	1933 (12)	1936 (12)	1946 (6)	1947 (6)
Age at first event	71 $(12{)}^{*}$	68 (12)	65 $(7{)}^{*}$	64 (8)
Diabetes (%)	21	18	19	19
Hypertension (%)	59	57	67	$70^{*}$
BMI	28.2 (4.4)	28.3 (4.6)	29.0 (5.3)	28.9 (5.2)
Smoking (%)	62	64	66	66
Cholesterol	5.73 $(1.33{)}^{*}$	5.72 (1.21)	5.6 (1.2)	5.6 (1.2)
Systolic BP	144 $(24{)}^{*}$	141 (22)	142 (19)	141 (19)
Diastolic BP	79.4 (14.2)	79.0 (13.3)	82.6 (10.8)	82.5 (10.7)
	MI HUNT		MI UKBB	
	Single events	Recurrent events	Single events	Recurrent events
	n=1,651	n=1,615	n=6,584	n=1,615
Male (%)	70.7	67.2	78	78
Birth year	1935 (12)	1934 (13)	1947 (6)	1947 (6)
Age at first event	64 (11)	68 $(12{)}^{*}$	59 (8)	61 $(9{)}^{*}$
Diabetes (%)	19	$25^{*}$	21	$35^{*}$
Hypertension (%)	52	$58^{*}$	74	$88^{*}$
BMI	27.7 (4.0)	28.0 (4.1)	28.8 (4.5)	29.3 $(5.0{)}^{*}$
Smoking (%)	$76^{*}$	71	72	74
Cholesterol	5.67 (1.45)	5.92 $(1.44{)}^{*}$	5.50 (1.27)	5.45 (1.28)
Systolic BP	140 (22)	144 $(23{)}^{*}$	140 (20)	141 (20)
Diastolic BP	78.7 (12.9)	80.0 (13.4)	81.1 (11.0)	80.5 (11.0)

Continuous variables are presented as mean (SD), and dichotomous variables are reported in percentages. Values with asterisks indicate a significant difference between the single and recurrent group, where the asterisk denotes the larger value. Specifics of the applied statistical tests are given in the Methods section.

However, the reverse trends emerge when analyzing the MI groups in the HUNT study. Here, patients experiencing recurrent MI events are older (adjusted p-value $<10^{-16}$) and demonstrate higher rates of diabetes and hypertension (adjusted p-values $8.8\times 10^{-5}$ and $7.6\times 10^{-3}$, respectively), alongside elevated levels of cholesterol and systolic blood pressure (adjusted p-values $5.4\times 10^{-6}$ and $1.4\times 10^{-5}$, respectively). In addition, there is a tendency toward higher BMI and diastolic blood pressure within this group. These trends persist within the UKBB MI cohorts, where the recurrent MI event group exhibits higher age, BMI, and prevalence of diabetes and hypertension (adjusted p-values $6\times 10^{-14}$, $3.9\times 10^{-5}$, $<10^{-16}$, and $<10^{-16}$, respectively) compared to the single MI event group. Moreover, there is a tendency toward higher systolic blood pressure levels within the UKBB single MI event group.

In summary, our observations reveal distinct patterns between patients experiencing single AF events and those with recurrent AF events. Notably, the single AF event group tends to be older at their initial event and exhibits worse health conditions and lifestyle factors compared to the recurrent AF group. Based on these findings, we hypothesize that single AF events may be primarily influenced by age and lifestyle factors, whereas recurrent AF events may be driven by genetic factors. The characteristics related to MI point in the opposite direction, since patients experiencing recurrent MI events are older and generally exhibit worse health conditions and lifestyle factors compared to those with only one MI event (and survive it). For MI, we therefore consider two alternative hypotheses: (i) recurrent MI events are associated with the age at the first event and worsened health conditions and single MI events are driven by genetic factors, or (ii) both single and recurrent MI events share common genetic factors, but recurring MI events are influenced by higher age and other lifestyle factors, affecting the risk of subsequent MI occurrences.

### 3.2 Genetic differences

In the following sections, we explore our hypotheses (as defined above) for AF and MI by investigating genetic differences between the groups identified through the GWAS meta-analyses.

#### 3.2.1 Genetic differences in AF

**Figure 1:**
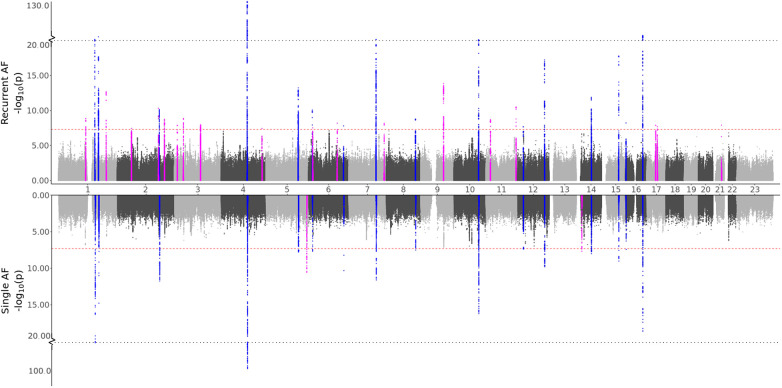
GWAS meta-analysis results for AF. Top: Comparing recurrent AF patients to AF-free controls. Bottom: Comparing single AF patients to AF-free controls. Blue spikes represent regions of SNPs found to be statistically significant in both GWAS studies (common), while magenta spikes represent statistically significant regions of SNPs specifically associated with the given AF group.

To test our hypothesis that patients experiencing recurrent AF events are more genetically susceptible than patients experiencing single AF events, we perform three GWAS meta-analyses (see Methods). The GWAS meta-analysis comparing single to recurrent AF events found no regions with significantly different effects. Some SNPs were identified to be of genome-wide significance in the HUNT population, but these were rare variants (MAF ≤0.2%), and we removed them through filtering prior to the meta-analysis. Comparing the GWAS meta-analyses of each group against healthy controls (Table 2 and Figure 1), we find that 18 regions are specifically associated with recurrent AF events: 2 are specifically associated with single AF events and 16 are identified in both GWAS investigations. Many regions comprise multiple SNPs that exhibit significant effects in only one of the study groups. Five regions identified in the recurrent AF GWAS study consist of only one SNP, yet these are identified with similarly strong effects in both the HUNT and the UKBB studies, indicating a genuine association. Regional plots of the single SNP hits uniquely associated with recurrent AF are shown in Supplementary File 1, Figures S15–S19. The presented results show that more than half of the identified regions are specifically linked to single or recurrent AF, supporting the hypothesis that patients who have experienced recurrent AF events are genetically more susceptible than those who have only experienced one event and survived it.

**Table 2:** AF variants found to be significant in the GWAS meta-analysis.

AFull	AF study	Known	rsID	Chr:pos_Ref/Alt	Gene	Function	Effect	StdErr	p-value	Dir	Nsnps
Yes	Common	Yes	rs6691463	1:154814538_C/G	*KCNN3*	Intronic	−0.16	0.015	$3.5\times 10^{-24}$	−−	166
Yes	Common	Yes	rs72700114	1:170193825_G/C	*NTMT2*	Intergenic	0.36	0.031	$9.1\times 10^{-32}$	++	239
Yes	Common	Yes	rs1429094	2:179515774_A/G	*TTN*	Intergenic	−0.14	0.02	$1.8\times 10^{-12}$	−−	64
Yes	Common	Yes	rs6843082	4:111718067_G/A	*PITX2*	Intergenic	−0.5	0.021	$2.4\times 10^{-132}$	−−	553
Yes	Common	Yes	rs678897	5:137441065_G/C	*NME5*	Intergenic	0.13	0.017	$5.7\times 10^{-14}$	++	229
Yes	Common	Yes	rs112899072	6:16417852_C/G	*ATXN1*	Intronic	0.15	0.023	$8.6\times 10^{-11}$	++	10
Yes	Common	Yes	rs117984853	6:149399100_G/T	*UST*	Downstream	0.16	0.025	$4.9\times 10^{-11}$	++	2
Yes	Common	Yes	rs11773845	7:116191301_C/A	*CAV1*	Intergenic	0.16	0.016	$4\times 10^{-24}$	++	218
Yes	Common	Yes	rs17337621	8:124542519_G/C	*FBXO32*	Intronic	0.19	0.032	$1.7\times 10^{-9}$	++	5
Yes	Common	Yes	rs1389189	10:105486077_A/G	*SH3PXD2A*	Intronic	−0.14	0.017	$6.1\times 10^{-17}$	−−	122
Yes	Common	Yes	rs137913153	12:24776752_A/G	*SOX5*	Intergenic	0.11	0.02	$4.2\times 10^{-8}$	++	8
Yes	Common	Yes	rs883079	12:114793240_C/T	*TBX5*	UTR3	0.15	0.017	$5.8\times 10^{-18}$	++	67
Yes	Common	Yes	rs1152591	14:64680848_A/G	*SYNE2*	Intronic	0.11	0.015	$1.3\times 10^{-12}$	++	67
Yes	Common	Yes	rs7172038	15:73667255_T/G	*HCN4*	Intergenic	−0.18	0.021	$1.6\times 10^{-18}$	−−	44
Yes	Common	Yes	rs140185678	16:2003016_G/A	*RPL3L*	Exonic	0.25	0.043	$6.1\times 10^{-9}$	++	1
Yes	Common	Yes	rs4499262	16:73059159_C/A	*ZFHX3*	Intronic	0.26	0.021	$2.5\times 10^{-35}$	++	108
Yes	Recurrent	Yes	rs9428227	1:116309200_C/T	*CASQ2*	Intronic	0.093	0.015	$1.3\times 10^{-9}$	++	24
Yes	Recurrent	Yes	rs2270543	1:203030685_T/C	*PPFIA4*	Intronic	0.11	0.015	$2\times 10^{-13}$	++	26
Yes	Recurrent	Yes	rs12463885	2:61457996_A/C	*USP34*	Intronic	0.086	0.016	$3.6\times 10^{-8}$	++	1
Yes	Recurrent	Yes	rs56181519	2:175555714_C/T	*WIPF1*	Intergenic	−0.12	0.018	$4.8\times 10^{-11}$	−−	4
Yes	Recurrent	Yes	rs7605146	2:201183888_G/A	*SPATS2L*	Intronic	0.094	0.016	$1.8\times 10^{-9}$	++	40
Yes	Recurrent	Yes	rs4642101	3:12842223_T/G	*CAND2*	Intronic	−0.091	0.016	$1.3\times 10^{-8}$	−−	2
Yes	Recurrent	Yes	rs6790396	3:38771925_C/G	*SCN10A*	Intronic	−0.095	0.016	$1.4\times 10^{-9}$	−−	16
Yes	Recurrent	Yes	rs73228569	3:111614052_T/C	*PHLDB2*	Intronic	0.12	0.021	$1.5\times 10^{-8}$	++	34
Yes	Recurrent	Yes	rs12646050	4:174634261_A/G	*RANP6*	Intergenic	0.14	0.026	$3.7\times 10^{-8}$	++	1
Yes	Recurrent	Yes	rs34969716	6:18210109_G/A	*KDM1B*	Intronic	0.095	0.017	$3.1\times 10^{-8}$	++	1
Yes	Recurrent	Yes	rs2684249	6:122392511_T/C	*HSF2*	Intergenic	0.09	0.016	$6.4\times 10^{-9}$	++	1
No	Recurrent	Yes	rs758890	7:150655643_G/A	*KCNH2*	Intronic	−0.094	0.016	$7\times 10^{-9}$	−−	5
Yes	Recurrent	Yes	rs10993463	9:97807233_C/T	*AOPEP*	Intronic	0.12	0.016	$1.3\times 10^{-14}$	++	88
Yes	Recurrent	Yes	rs2568119	11:20004957_G/A	*NAV2*	Intronic	0.11	0.018	$2\times 10^{-9}$	++	26
Yes	Recurrent	Yes	rs3765618	11:128769876_C/G	*KCNJ5*	UTR3	−0.18	0.027	$3.2\times 10^{-11}$	−−	12
Yes	Recurrent	Yes	rs12944882	17:37983492_T/C	*IKZF3*	Intronic	0.088	0.015	$1.2\times 10^{-8}$	++	2
Yes	Recurrent	Yes	rs17608766	17:45013271_T/C	*GOSR2*	UTR3	−0.12	0.022	$2.1\times 10^{-8}$	−−	2
Yes	Recurrent	Yes	rs2834618	21:36119111_T/G	*CLIC6*	Intergenic	0.15	0.026	$1.2\times 10^{-8}$	++	1
Yes	Single	Yes	rs1223535129	5:172664353_CG/C	*NKX2-5*	Intergenic	0.11	0.017	$2.7\times 10^{-11}$	?+	31
Yes	Single	Yes	rs28631169	14:23888183_C/T	*MYH7*	Intronic	0.1	0.018	$2.1\times 10^{-8}$	++	2

The lead variant (lowest p-value) for each independent region is listed. The “AFfull” column describes if the variant (or any of the significant variants in this region) is also identified in the full AF GWAS meta-analysis. The “AF study” column shows in which of the studies the variant/region was found to be significant (“common” meaning significant in both the single and the recurrent GWAS meta-analysis). The “Known” column reports if this region is previously known for AF association. The subsequent columns are “rsID,” position, nearest gene, and the function of the lead SNP as well as effect size, standard error, and p-value from the meta-analysis. “Dir” corresponds to the direction of the effect in the HUNT and UKBB GWAS meta-analysis (an entry of ? means not included in the meta-analysis—see Supplementary File 4 for allele frequencies)—and “Nsnps” shows the number of significant SNPs in the region.

All regions had previously been associated with AF, and all regions except one (chromosome 7, in the *KCNH2* gene) were identified in the full AF GWAS meta-analysis by comparing all AF cases against healthy controls. This indicates that all the regions identified as unique for single or recurrent AF (excluding the *KCNH2* gene region) have an effect when compared to healthy controls, with the true effect being mainly or solely for patients experiencing single or recurrent AF. The five SNPs in the *KCNH2* gene, however, are not detected in our full GWAS meta-analysis and therefore only show an effect for patients experiencing recurrent AF.

To our knowledge, only seven genes have previously been found to be associated with AF recurrence: *SOX5, CAV1, EPHX2, ITGA9, SLC8A1, TBX5*, and *PITX2* (1, 8, 10, 37). Our findings show that regions proximate to the *SOX5, CAV1, TBX5*, and *PITX2* genes are identified in both the single and the recurrent GWAS, yielding comparable effects. Thus, there is no evidence of differences in the impact of these regions between the two groups. Furthermore, no regions were identified near the *EPHX2, SCL8A1*, and *ITGA9* genes. Variants near the *NAV2* and *SCN10A* genes have previously been tested for their effect in recurrent AF events without any significant findings (37, 38). In this study, we discovered 26 and 16 SNPs located within and nearby the NAV2 and SCN10A genes, respectively, that are exclusively associated with recurrent AF, suggesting that these SNPs have a distinct effect on recurrent AF patients compared to single AF cases.

Several of the genes listed in Table 2 code for functions related to AF. Two of the genes listed as “Common” (*KCNN3* and *HCN4*) and three genes identified uniquely for recurrent AF (*SCN10A, KCNH2*, and *KCNJ5*) are related to electrophysiological activity, coding for potassium and sodium channels. Other genes listed as “Common” in Table 2 code for functions directly linked to heart activity and AF (*TTN, TBX5, SYNE2*, and *RPL3L*) , or they are indirectly linked to AF through comorbidities (*ATXN1, CAV1, SH3PXD2A*, and *ZFHX3*). Two of the recurrent AF genes also code for functions directly or indirectly linked to AF (*CASQ2* and *GOSR2*), and some genes indicate a possible indirect link related to comorbidities, e.g., hypertension or malignancy (*PPFIA4, USP34, WIPF1, SPATS2L, CAND2*, and *AOPEP*). The two genes uniquely identified for single AF events have been shown important for myocardial diseases and cardiac abnormalities, coding for functions found to be central in malformation in heart (*NKX2-5*) and myosin (*MYH7*).

The genetic observed scale heritability was found to be 0.0139 (SE 0.0024) for recurrent AF and 0.0086 (SE 0.0018) for single AF events.

#### 3.2.2 Genetic differences in MI

**Figure 2:**
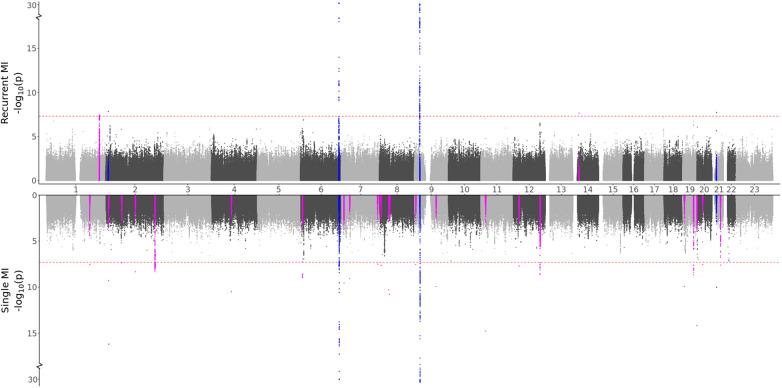
GWAS meta-analysis results for MI. Top: Comparing recurrent MI patients to MI-free controls. Bottom: Comparing single MI patients to MI-free controls. Blue spikes represent regions of SNPs found to be statistically significant in both GWAS studies (common), while magenta spikes represent statistically significant regions of SNPs that are specifically associated with the given MI group.

Based on the characteristics of the two MI groups, we formulated two hypotheses: (i) Recurrent MI events are associated with the age at the first event and worsened health conditions and single MI events are driven by genetic factors, or (ii) Both single and recurrent MI events share common genetic factors, but recurring MI events are influenced by higher age and other lifestyle factors, affecting the risk of subsequent MI occurrences. Testing for direct genetic differences between the two MI groups, the GWAS meta-analysis (comparing single to recurrent events) did not detect any regions with significant effects. When testing for genetic effects in each group as compared to MI-free controls, the GWAS meta-analyses shown in Figure 2 and Table 3 identified four regions that are in common for both groups, 24 regions that are specifically identified for the single event group, and two regions that are unique for the recurrent events group. Hence, some genetic factors are common for both groups, but most identified genetic effects are unique to patients experiencing only one event of MI and surviving it. These results are in support of our first hypothesis.

**Table 3:** MI variants that are significant in the GWAS meta-analysis.

MIfull	MI study	Known	rsID	Lead	Gene	Function	Effect	StdErr	p-value	Dir	Nsnps
Yes	Common	No*	rs7600627	2:10543580_T/G	*HPCAL1*	Intronic	0.41	0.049	$6.5\times 10^{-17}$	+?	1
Yes	Common	Yes	rs55730499	6:161005610_C/T	*LPA*	Intronic	0.3	0.031	$1.4\times 10^{-21}$	++	59
Yes	Common	Yes	rs1333049	9:22125503_G/C	*CDKN2A*	Intergenic	0.18	0.016	$3.8\times 10^{-27}$	++	184
Yes	Common	No	rs2846882	21:18930412_T/A	*CXADR*	Intronic	−0.29	0.044	$9.6\times 10^{-11}$	−?	1
No	Single	No	rs72720974	1:180559567_A/G	*OVAAL*	Intergenic	0.23	0.042	$3\times 10^{-8}$	+?	1
No	Single	No*	rs1620983	2:10028920_T/G	*TAF1B*	Intronic	0.27	0.043	$5.3\times 10^{-10}$	+−	1
Yes	Single	No*	rs2422293	2:65033227_A/G	*SERTAD2*	Intergenic	0.26	0.047	$4.4\times 10^{-8}$	+?	1
No	Single	No*	rs34337514	2:121458109_A/T	*GLI2*	Intergenic	0.22	0.038	$4.7\times 10^{-9}$	+−	1
Yes	Single	Yes	rs147932234	2:203951846_T/C	*NBEAL1*	Intronic	−0.15	0.025	$4.8\times 10^{-9}$	−−	254
Yes	Single	No	rs2868125	4:81955408_T/G	*BMP3*	Intronic	0.27	0.041	$3.2\times 10^{-11}$	+?	1
Yes	Single	No	rs576614613	6:7442904_T/C	*RIOK1*	Intergenic	−0.26	0.043	$1.1\times 10^{-9}$	−?	5
Yes	Single	No	rs34945893	7:9691418_A/T	*AC096553.5*	Intergenic	0.26	0.041	$2.9\times 10^{-10}$	+?	1
No	Single	No*	rs1544558	7:33508624_T/G	*BBS9*	Intronic	0.26	0.042	$8.8\times 10^{-10}$	+?	1
Yes	Single	Yes	rs3918226	7:150690176_C/T	*NOS3*	Intronic	0.17	0.03	$3.2\times 10^{-8}$	++	1
No	Single	No	rs7016007	8:5781499_G/T	*MCPH1*	Intergenic	−0.28	0.05	$2.4\times 10^{-8}$	−?	1
Yes	Single	No	rs56097134	8:37067371_G/A	*KCNU1*	Intergenic	−0.24	0.037	$5\times 10^{-11}$	−−	1
Yes	Single	No*	rs1472790078	8:41141915_A/G	*SFRP1*	Intronic	0.28	0.042	$1.7\times 10^{-11}$	+?	1
No	Single	No	rs10814885	9:4209824_C/G	*GLIS3*	Intronic	0.24	0.044	$2.9\times 10^{-8}$	+?	1
Yes	Single	No	rs111991434	9:89155924_A/G	*TUT7*	Intergenic	0.28	0.043	$1.2\times 10^{-10}$	+?	1
Yes	Single	No*	rs58771640	11:19767929_T/A	*NAV2*	Intronic	−0.34	0.043	$1.7\times 10^{-15}$	−?	1
Yes	Single	No*	rs4625573	12:24378427_T/C	*SOX5*	Intronic	0.27	0.048	$2\times 10^{-8}$	+?	1
Yes	Single	Yes	rs1876263690	12:111907431_A/AC	*ATXN2*	Intronic	0.098	0.016	$2.4\times 10^{-9}$	++	10
No	Single	Yes	rs77215829	12:112618346_A/C	*HECTD4*	Intronic	0.14	0.024	$7.8\times 10^{-9}$	++	2
No	Single	No*	rs2352955	19:7152404_A/G	*INSR*	Intronic	0.23	0.035	$1.2\times 10^{-10}$	++	1
Yes	Single	Yes	rs55766194	19:45013423_A/G	*TOMM40*	Intronic	0.29	0.048	$2.2\times 10^{-9}$	+?	9
Yes	Single	No	rs6076475	20:360933_A/C	*TRIB3*	Upstream	0.41	0.052	$6.7\times 10^{-15}$	+?	1
No	Single	No	rs1569677	20:24404965_A/G	*SYNDIG1*	Intergenic	0.22	0.04	$3\times 10^{-8}$	+?	1
Yes	Single	Yes	rs28451064	21:35593827_G/A	*MRPS6*	Intergenic	0.14	0.024	$2.5\times 10^{-8}$	++	1
Yes	Recurrent	Yes	rs46i18978	1:222779187_C/G	*MIA3*	Intergenic	−0.15	0.028	$3.6\times 10^{-8}$	−−	5
Yes	Recurrent	No	rs59875208	14:26557764_C/A	*NOVA1*	Intergenic	−0.23	0.042	$2.2\times 10^{-8}$	−−	1

The lead variant (lowest p-value) for each independent region is listed. The “MIfull” column describes if the given variant (or any of the significant variants in this region) is also identified in the full MI GWAS meta-analysis. The “MI study” column shows in which of our studies the variant/region was found to be significant (an entry of “common” means that it was present in both the single and the recurrent GWAS meta-analysis). The “Known” column shows if this region is previously related to MI (“Yes”), a relevant CVD trait (“No*”), or neither (“No”). The next columns are “rsID,” “nearest gene,” “the function of the lead SNP,” “effect size,” “standard error,” and “p-value” from the meta-analysis. “Dir” corresponds to the direction of the effect in the HUNT and UKBB GWAS meta-analysis (a value “?” indicates that it was not included in the meta-analysis—see Supplementary File 4 for allele frequencies)—and “Nsnps” shows the number of significant SNPs in the region.

Some distinct regions, including the SNPs in the *NBEAL1* and *ATXN2* genes for single MI events and SNPs in the *MIA3* gene for recurrent events, exhibit substantial effects for multiple SNPs in the region, with comparable effects in both the HUNT and UKBB populations. Several regions represent suggestive findings comprising only single SNPs and are only identified in the HUNT population (regional plots of the single SNP hits uniquely associated with single or recurrent MI are shown in Supplementary File 1, Figures S20–S39). However, as shown in Supplementary File 4, these variants are not HUNT-specific since they are reported with relatively high frequencies in the general European population. Hence, although they are rare in the UKBB population and thereby not included in the meta-analysis, including a different European study population could validate or dispute the effect identified here. Also, many of these regions are well-known for MI, further suggesting that these findings might be valid.

Comparing the GWAS meta-analysis of all MI cases to MI-free controls, we find that all four regions that were identified as common for single and recurrent MI (regions in or close to the genes *HPCAL1, LPA, CDKN2A*, and *CXADR*) were also found in the full MI GWAS meta-analysis. The two regions that were specifically associated with recurrent MI events (regions close to the *MIA3* and *NOVA1* genes) were also identified in the full MI GWAS, but nine of the regions specifically associated with single MI were not detected in the full MI GWAS (regions in or close to the genes *OVAAL, TAF1B, GLI2, BBS9, MCPH1, GLIS3, HECTD4, INSR*, and *SYNDIG1*). Hence, certain regions identified in the full MI GWAS are exclusively linked to either single or recurrent MI, and some regions are only observed when patients with single MI events are filtered out, emphasizing the need for sub-dividing the MI groups.

We notice that 24 regions are specifically associated with a single MI. Among these, 10 regions, proximal to or within the genes *OVAAL, BMP3, RIOK1, AC096553.5, MCPH1, KCNU1, GLIS3, TUT7, TRIB3*, and *SYNDIG1*, represent novel associations with MI and have not been previously linked to Cardiovascular disease (CVD)-related traits. These regions are predominantly characterized by a single SNP, with the exception of five SNPs in proximity to the *RIOK1* gene. These SNPs are only identified within the HUNT population, barring the SNP near the *KCNU1* gene. Interestingly, some of these regions encode for functions similar to those of genes previously associated with MI. Three of these genes, namely, *OVAAL, RIOK1*, and *TUT7*, are commonly associated with malignancy, akin to *NBEAL1*, where we identified a known MI region comprising 254 SNPs uniquely associated with single MI. Other genes encode proteins involved in calcium handling (*BMP3* and *SYNDIG1*) or are associated with diabetes mellitus (*GLIS3* and *TRIB3*), suggesting a potential link to accelerated atherosclerosis development. Similarly, both *ATXN2* and *HECTD4* are associated with diabetes mellitus, and we identified known MI regions uniquely associated with single MI in these genes. Two regions exclusively linked to recurrent MI events were identified, both exhibiting negative effects in the HUNT and the UKBB population. The region near the *MIA3* gene has been previously associated with MI, while the single SNP near the *NOVA1* gene, which may also be related to malignancy, represents a novel finding. Collectively, these findings underscore the potential relevance of these genes to MI. Further investigations are warranted to ascertain if these effects are replicable in other European and non-European populations and to determine the specific links of these SNPs/genes to MI, particularly in relation to single or recurrent MI events.

The observed genetic scale heritability was found to be 0.0051 (SE 0.0011) for single MI and 0.0039 (SE 0.0011) for recurrent MI.

### 3.3 Identification of additional phenotypes affected by SNPs through PheWAS

To delve deeper into the genetic distinctions observed between single and recurrent AF and MI, we conducted a PheWAS analysis. This enabled us to pinpoint other phenotypes associated with the same set of SNPs designated as either common or unique for single and recurrent AF and MI.

**Figure 3:**
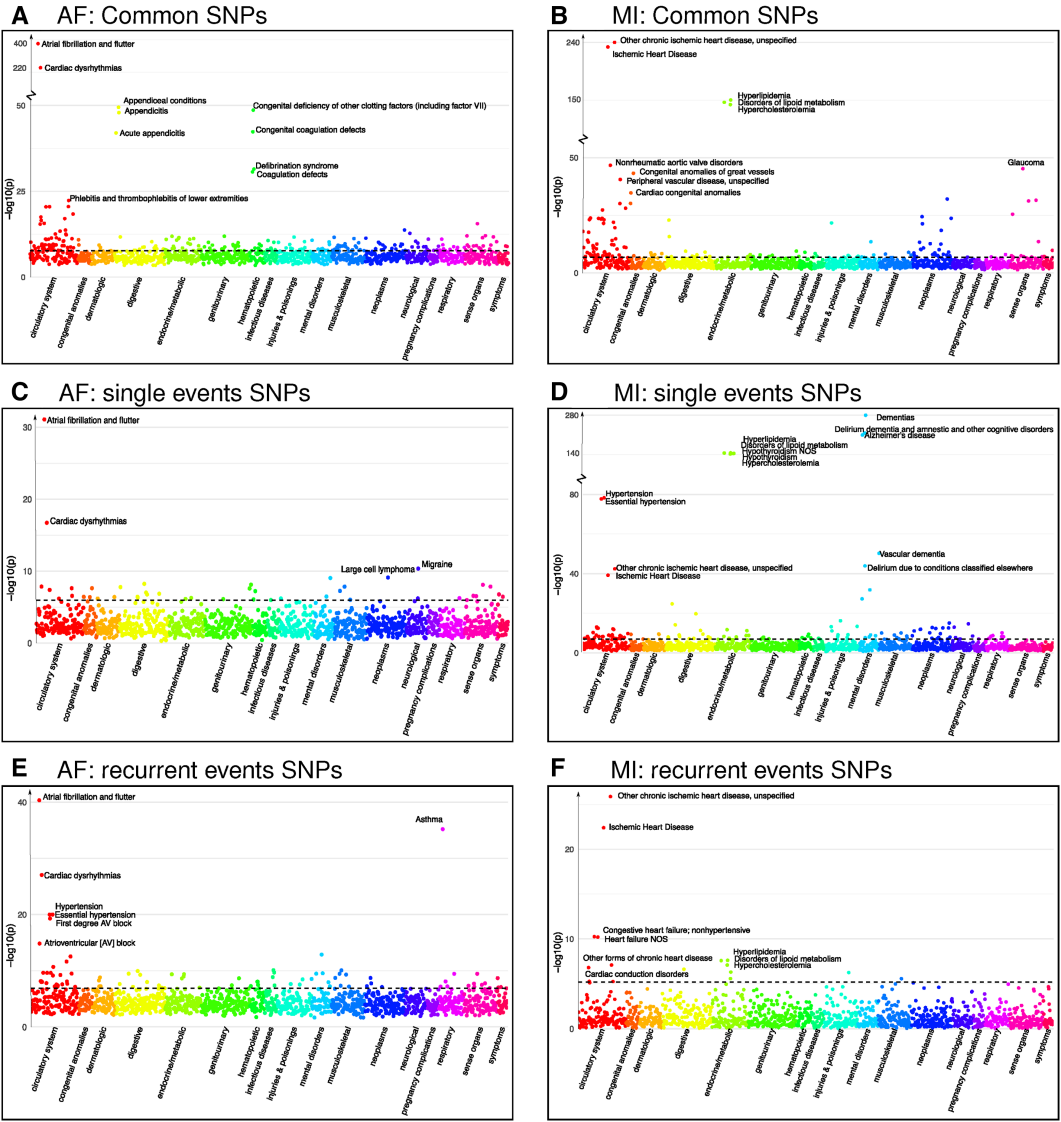
Phenocodes associated with each set of SNPs found for both single and recurrent AF/MI or uniquely for one of them. The x-axis shows each of the 1,326 phenocodes sorted by phenocode category, and the y-axis shows the lowest p-value for the association between the phenocode and the SNPs in the set. The dotted line shows the threshold for significant associates, which vary according to the number of SNPs in each set. (**A**) A set of 1,903 SNPs found in common for both single and recurrent AF. (**B**) A set of 245 SNPs found in common for both single and recurrent MI. (**C**) A set of 33 SNPs found uniquely for single AF events. (**D**) A set of 299 SNPs found uniquely for single MI events. (**E**) A set of 286 SNPs found uniquely for recurrent AF events. (**F**) A set of six SNPs found uniquely for recurrent MI events.

Our PheWAS investigation of the SNPs identified as common for both single and recurrent AF revealed a total of 1,903 SNPs linked with 236 phenocodes (shown in Figure 3 and Supplementary File 2). Not surprisingly, the strongest associations were found for *Atrial fibrillation and flutter* and *Cardiac dysrhythmia* (p-value $10^{-400}$ and $10^{-220}$). Furthermore, we identified robust associations with phenocodes related to *Appendiceal conditions* and *Coagulation defects*. Notably, the circulatory system category emerged as the predominant category, encompassing 54 phenocodes. This includes, but is not limited to, conditions such as *Phlebitis and thrombophlebitis, Sinoatrial node dysfunction (Bradycardia), Heart failure*, and *Hypertension*.

Intriguingly, the two identified regions specifically associated with single AF consist of 33 SNPs that exhibit significant association with 44 phenocodes (see Supplementary File 2). Not surprisingly, the strongest associations for these SNPs pertain to the phenocodes *Atrial fibrillation and flutter* and *Cardiac dysrhythmias*, the remaining 42 phenocodes span a diverse array of phenocode categories. These include not only *Migraine* and *Large cell lymphoma* but also conditions such as *Arrhythmia (cardiac) NOS, Paroxysmal supraventricular tachycardia*, and *Cerebral atherosclerosis*.

In contrast, the 18 regions specifically associated with recurrent AF events consisting of 286 SNPs show a significant association with 91 phenocodes (see Supplementary File 2), and a majority of the strong associations pertain to phenocodes of the circulatory system category. Again, the phenocode with the most potent associations are *Atrial fibrillation and flutter* and *Cardiac dysrhythmias*. In addition, these SNPs also display significant associations with *Asthma* and 27 phenocodes from the circulatory system, including conditions such as *Hypertension, Atrioventricular block, Cardiomyopathy, Heart failure, Ischemic heart disease, Cardiac arrest*, and *Palpitations*. Collectively, these results underscore genetic susceptibility disparities between patients experiencing single vs. recurrent AF events. In particular, SNPs specifically tied to recurrent AF are linked to a broad range of phenocodes related to the heart and circulatory system, in contrast to SNPs exclusively linked to single AF events.

Regarding MI, we identified four regions associated with both single and recurrent MI, comprising 245 SNPs that exhibit significant associations to 144 phenocodes (see Supplementary File 2). The most prominent associations are observed with *Ischemic heart disease* and *Hyperlipidemia* disorders. In addition, numerous diseases within the circulatory system category, such as *Non-rheumatic aortic valve disorders, Peripheral vascular disease, Stricture of artery, Hypertension*, and *Heart valve disorders*, are also strongly associated.

The 24 regions specifically identified for single MI events consist of 299 SNPs that are associated with 128 phenocodes (see Supplementary File 2). These include *Ischemic heart disease, Hypertension*, and diseases of *Hyperlipidemia*. In addition, there are strong associations with neurodegenerative disorders, such as *Dementia, Alzheimer’s*, and *Delirium*. These SNPs are furthermore linked with 33 phenocodes from the circulatory system category, highlighting conditions such as *Cerebral ischemia, Cardiac conduction disorders, Heart failure, Aortic valve disease*, and *Pulmonary heart disease*.

Notably, the two regions consisting of six SNPs specifically identified for recurrent MI were associated with a mere 16 phenocodes (see Supplementary File 2). While these included *Ischemic heart disease, Heart failure, Cardiac conduction disorders*, and diseases of *Hyperlipidemia*, they lacked the other 27 circulatory system disorders identified for the single MI SNPs. Once again, these findings emphasize the genetic differences between patients experiencing single and recurrent MI. SNPs specifically associated with single MI events appear to be associated with a broader and more diverse range of cardiovascular disorders compared to those solely linked to recurrent MI.

### 3.4 Gene sets and co-expression network neighborhood

In our final analysis, we leverage multiple sets of gene expression data from the GTEx consortium (32) measured in tissue sub-types taken from the heart, muscle, skeletal, artery, and kidney to generate gene co-expression networks (see Methods for details). Here, our expectation is that highly correlated genes have a regulatory relationship or similar response in a condition (31). Thus, this approach should uncover genes that display an expression profile that most closely links to the set of target genes found through our GWAS analyses, and we investigate their functions.

#### 3.4.1 AF-associated genes in co-expression networks

Differential gene expression analysis of the 18 genes identified in recurrent AF (listed as Recurrent in Table 2) reveals a significant upregulation of these genes in atrial appendage tissues from the heart. Furthermore, elevated expression levels are discerned in left ventricular heart, artery tibial, and skeletal muscle tissues (see Supplementary File 1, Figure S9). Gene ontology analysis indicates that this set of genes is significantly enriched for cell–cell signaling involved in cardiac conduction (fold enrichment (FE) >100, FDR $=1.17\times 10^{-2}$), cardiac muscle cell action potential (FE =70.03, FDR $=3.15\times 10^{-2}$), and regulation of heart rate (FE =43.99, FDR $=1.62\times 10^{-2}$).

**Figure 4:**
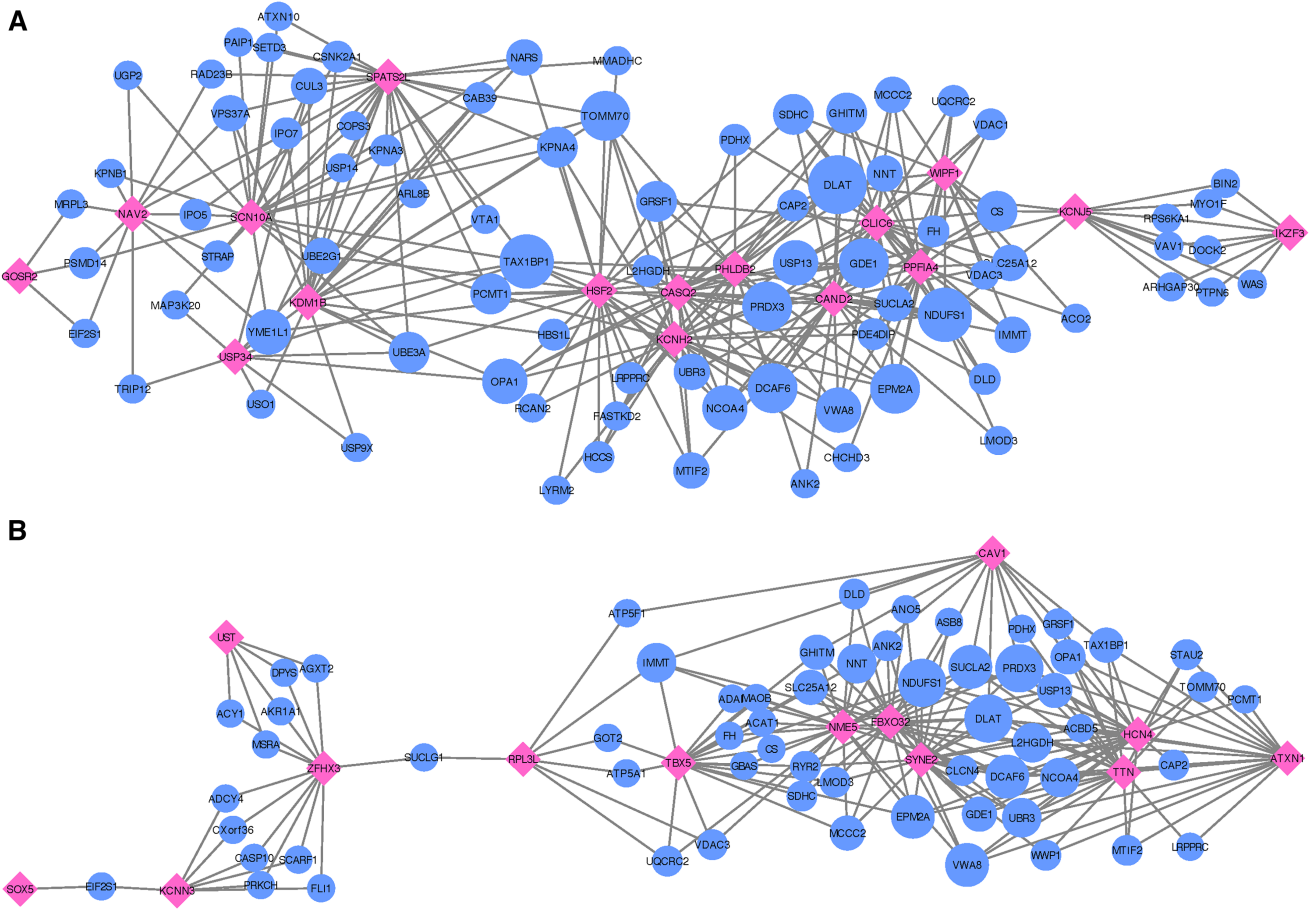
Networks showing the strongest shared neighborhood of co-expressed genes for the GWAS (target) genes associated with (**A**) recurrent AF uniquely and (**B**) both single and recurrent AF. Pink diamond nodes represent the target genes and blue circular nodes represent the neighboring genes. The sizes of the blue nodes are scaled according to their number of nearest neighbors in the network.

Following the co-expression analysis approach detailed in the Methods section, we find that 16 of the 18 recurrent AF genes show strong co-expression with other genes in heart, artery, kidney, and skeletal muscle tissues. Selecting the top 25 genes with the strongest connection to each of the 16 target genes, Figure 4A shows that all of the 16 target genes are connected through 82 shared neighboring genes (see Supplementary File 3), i.e., the 82 shared genes are among the top 25 strongest connections for two or more of the target genes. These 82 neighboring genes are significantly enriched for a variety of biological processes, including acetyl-CoA biosynthetic process from pyruvate (FE >100, FDR $=4.41\times 10^{-3}$), tricarboxylic acid cycle (FE =54.70, FDR $=1.38\times 10^{-6}$), NLS-bearing protein import into nucleus (FE =50.99, FDR $=1.93\times 10^{-3}$), inner mitochondrial membrane organization (FE =32.73, FDR $=9.49\times 10^{-4}$), respiratory electron transport chain (FE =10.72, FDR $=4.32\times 10^{-2}$), regulation of proteasomal protein catabolic process (FE =7.49, FDR $=4.94\times 10^{-2}$), proteasome-mediated ubiquitin-dependent protein catabolic process (FE =5.58, FDR $=3.79\times 10^{-2}$), and regulation of cellular catabolic process (FE =4.01, FDR $=9.85\times 10^{-3}$).

Focusing on the two genes specific to single AF events (listed as Single in Table 2), our analysis reveals that these genes show significant upregulation in left ventricle tissues of the heart and also high expression levels for atrial appendage tissues of the heart (see Supplementary File 1, Figure S10). Gene ontology analysis confirms that these genes are closely linked to adult heart development (FE >100, FDR $=7.77\times 10^{-3}$), ventricular cardiac muscle tissue morphogenesis (FE >100, FDR $=4.17\times 10^{-2}$), myofibril assembly (FE >100, FDR $=2.98\times 10^{-2}$), cardiac muscle contraction (FE >100, FDR $=2.47\times 10^{-2}$), and regulation of striated muscle contraction (FE >100, FDR = $2.87\times 10^{-2}$). Thus, although both target genes exhibit the specified enriched functions, an egocentric network analysis reveals that they do not share mutual genes with strong co-expression across the heart, artery, kidney, and skeletal muscle tissues. Therefore, while they may have functional overlap, the co-expressing gene partners diverge for each target gene.

In our comparative analysis of gene sets uniquely associated with either single or recurrent AF, we also evaluated genes that were consistent across both AF categories. Among the 16 genes identified for both AF groups (labeled as *Common* in Table 2), there was a significant upregulation in tissues of the heart’s left ventricle, atrial appendage, and skeletal muscles (see Supplementary File 1, Figure S11). These genes did not show any enrichment in specific gene ontology categories. Upon conducting an egocentric network analysis, we found that 14 out of these 16 genes displayed strong co-expression patterns with genes differentially expressed in the heart, arteries, kidneys, and skeletal muscles. Moreover, 13 of these genes were interconnected via 61 shared neighbor genes (see Figure 4B and Supplementary File 3). The 61 genes found in common for the 13 target genes show significant enrichment for fumarate metabolic process (FE >100, FDR $=1.83\times 10^{-2}$), regulation of atrial cardiac muscle cell action potential (FE >100, FDR $=2.51\times 10^{-2}$), regulation of mitochondrial RNA catabolic process (FE >100, FDR $=3.36\times 10^{-2}$), mitochondrial acetyl-CoA biosynthetic process from pyruvate (FE >100, FDR = $3.32\times 10^{-2}$), succinyl-CoA catabolic process (FE >100, FDR $=4.10\times 10^{-2}$), tricarboxylic acid cycle (FE =73.80, FDR $=5.34\times 10^{-8}$), branched-chain amino acid catabolic process (FE =46.69, FDR $=1.11\times 10^{-2}$), inner mitochondrial membrane organization (FE =35.33, FDR $=2.16\times 10^{-3}$), aerobic electron transport chain (FE =15.20, FDR $=2.98\times 10^{-2}$), mitochondrial ATP synthesis coupled electron transport (FE =14.37, FDR $=3.50\times 10^{-2}$), and alpha-amino acid catabolic process (FE =14.21, FDR $=3.53\times 10^{-2}$).

Thus, the gene sets identified as common and unique for single and recurrent AF events are upregulated in atrial appendage tissues of the heart, left ventricular tissues of the heart, and skeletal muscle tissues, where the gene set identified for recurrent AF and single AF events show significant upregulation in atrial appendage and left ventricular tissues of the heart, respectively, while the gene set identified for both single and recurrent AF shows significant upregulation of all three tissues. While both gene sets specifically identified for single and recurrent AF events show enrichment for functions related to heart and cardiac muscle, the recurrent AF gene set shows interesting enrichment of functions related to regulation of heart rate and cardiac muscle cell action potential. Collectively, these findings underscore the distinct genetic underpinnings between patients experiencing single vs. recurrent AF events, with the recurrent AF genes revealing more intricate processes.

#### 3.4.2 Network MI

The 24 nearest genes to the regions specifically identified for single MI events (listed as Single in Table 3) show no significant enrichment for any gene ontology terms. Differential gene expression analysis shows no significant up- or downregulation of these genes in any tissue, but significant expression levels in the tibial nerve and high expression levels in the tibial artery and aorta artery tissues (see Supplementary File 1, Figure S12).

**Figure 5:**
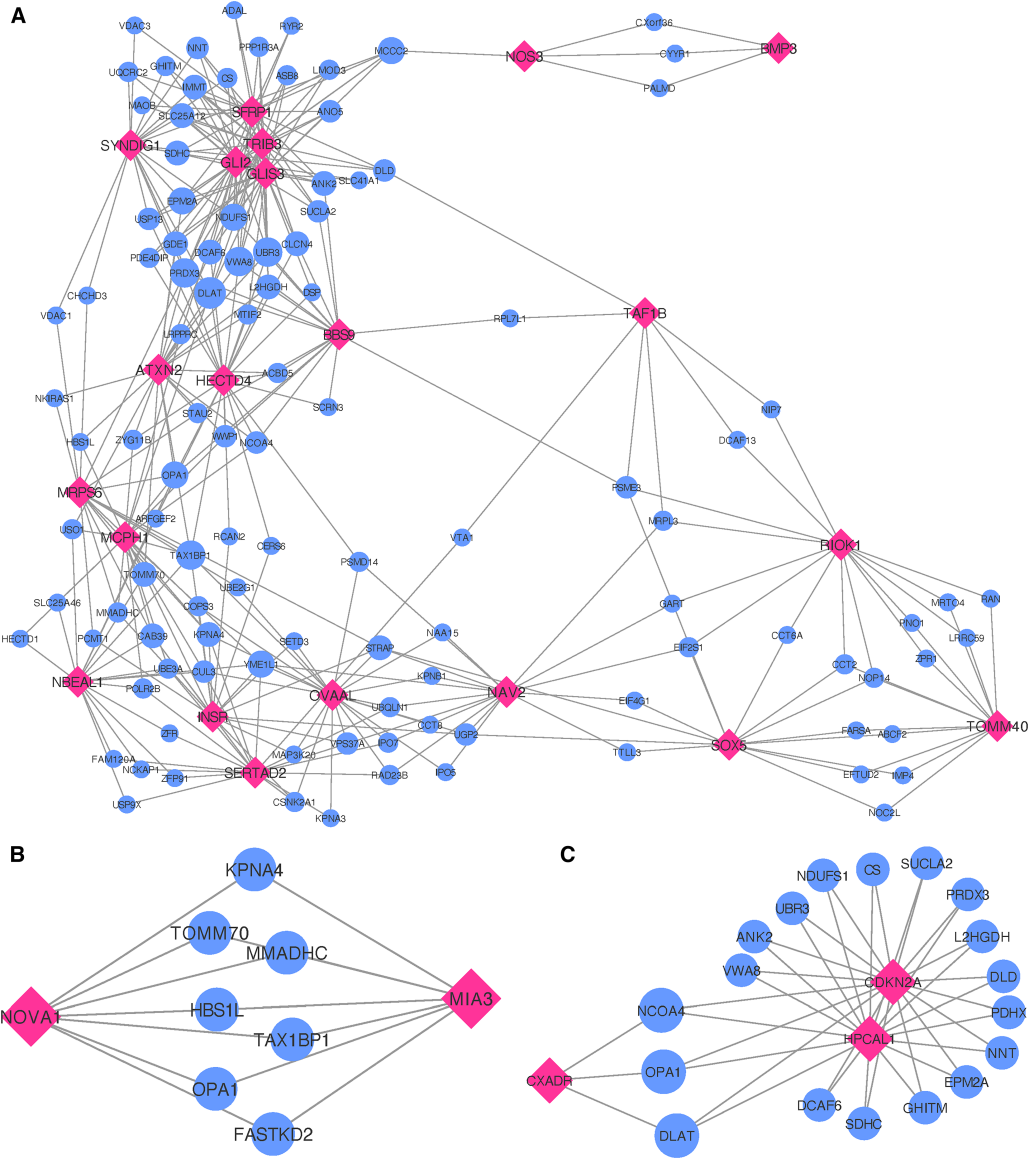
Networks showing the strongest shared neighborhood of co-expressed genes for the GWAS (target) genes associated with (**A**) single MI uniquely, (**B**) recurrent MI uniquely and (**C**) both single and recurrent MI. Pink diamond nodes represent the target genes and blue circular nodes represent the neighboring genes. The sizes of the blue nodes are scaled according to their number of nearest neighbors in the network.

When inspecting gene co-expression in heart, artery, kidney, and skeletal muscle tissues, we find that 21 of these genes show high co-expression with other genes in these tissues. Again, creating egocentric networks for each of these 21 target genes, we find that all 21 target genes cluster in a giant component, where 110 genes are connected to two or more of the target genes. The network depicted in Figure 5A illustrates genes that are interconnected with multiple single MI target genes. Notably, there is a dense cluster in the upper left portion of the figure, dominated by *GLI2, GLIS3, TRIB3*, and *SFRP1*, all of which are interconnected and share a majority of their neighborhood genes. The known associations of both *GLI2* and *SFRP1* with CVD-related traits, coupled with the detection of *TRIB3* and *SFRP1* in the comprehensive MI GWAS, suggest potentially shared functional roles of these four genes. Furthermore, they also exhibit significant interconnections with *BBS9, HECTD4, ATXN2*, and *SYNDIG1*. It is worth noting that *HECTD4* and *ATXN2* have recognized associations with MI.

Gene ontology analysis of the 110 genes shared between two or more target genes (see Supplementary File 3) show significant enrichment for positive regulation of several functional categories, more specifically establishment of protein localization to telomere (FE =54.18, FDR $=1.95\times 10^{-2}$), positive regulation of protein localization to the Cajal body (FE =49.26, FDR $=2.28\times 10^{-2}$), NLS-bearing protein import into nucleus (FE =38.02, FDR $=3.85\times 10^{-3}$), positive regulation of telomerase RNA localization to the Cajal body (FE =36.12, FDR $=4.17\times 10^{-2}$), cristae formation (FE $=36.12,4.00\times 10^{-2}$), tricarboxylic acid cycle (FE =29.13, FDR $=1.59\times 10^{-3}$), regulation of proteasomal protein catabolic process (FE =8.38, FDR $=1.63\times 10^{-3}$), regulation of ubiquitin-dependent protein catabolic process (FE =7.35, FDR $=2.27\times 10^{-2}$), proteasome-mediated ubiquitin-dependent protein catabolic process (FE =4.68, FDR $=4.76\times 10^{-2}$), purine-containing compound metabolic process (FE =4.21, FDR $=4.69\times 10^{-2}$), nucleotide metabolic process (FE =3.84, FDR $=4.69\times 10^{-2}$), and cellular component biogenesis (FE =2.06, FDR $=3.57\times 10^{-2}$).

Upon analyzing the two genes uniquely associated with recurrent MI (listed as Recurrent in Table 3), we observe neither significant enrichment in gene ontology terms nor any distinctive expression patterns across tissue types (see Supplementary File 1, Figure 13). In constructing egocentric networks for these genes, we identify seven overlapping genes (see Supplementary File 3) among the top 25 strongest correlations for each gene, as depicted in Figure 5B. While four of these seven neighboring genes (*TOMM70, FASTKD2, MMADHC*, and *OPA1*) are related to mitochondrial function and energy production, gene ontology analysis yielded no significant enrichment.

Investigating the genes that are common for both MI groups (listed as Common in Table 3), we find no significant differential co-expression in any of the tissues (see Supplementary File 1, Figure S14). No gene ontology terms were found to be significantly enriched for these four genes. The egocentric networks (Figure 5C) revealed that 3 of them are linked through 18 shared neighborhood genes (see Supplementary File 3). The 18 genes connecting the 3 target genes show significant enrichment for mitochondrial acetyl-CoA biosynthetic process from pyruvate (FE >100, FDR $=5.92\times 10^{-3}$), tricarboxylic acid cycle (FE >100, FDR $=5.37\times 10^{-7}$), negative regulation of release of cytochrome c from mitochondria (FE >100, FDR $=4.88\times 10^{-2}$), cell redox homeostasis (FE =81.70, FDR $=3.38\times 10^{-3}$), aerobic electron transport chain (FE =39.90, FDR $=2.07\times 10^{-2}$), mitochondrial ATP synthesis coupled electron transport (FE =37.71, FDR $=2.38\times 10^{-2}$), reactive oxygen species metabolic process (FE =32.68, FDR $=3.25\times 10^{-2}$), and mitochondrion organization (FE =12.85, FDR $=1.15\times 10^{-2}$).

In summary, MI gene sets do not exhibit notable tissue specificity or functional enrichment. Yet, the gene sets linked to both common and single MI incidents share multiple genes within their co-expression network neighborhoods. These shared genes exhibit functional enrichment for several pertinent processes. At the same time, the shared gene neighborhood specifically related to recurrent MI does not present any functional enrichment. However, some of the genes are related to mitochondrial function and energy production, similar to several of the enriched functions for the shared neighboring genes for common MI genes. Notably, the neighboring genes of the single MI genes show significant enrichment for several functions not identified for the recurrent of common neighboring gene sets. Only one of the biological processes identified in the neighborhood of single MI genes is also seen in the neighborhood of common genes. This suggests unique functions associated with genes specific to single MI incidents, shedding light on potential reasons why certain patients experience only one MI event.

## 4 Discussion

This study demonstrates distinct clinical characteristics and genetic predispositions between patients who experience a single AF/MI event and those with recurrent events. To ensure that the single AF and single MI patient groups represent relapse-free patients, we have applied a data filtering procedure to ensure that the ones who only have recorded single events were alive for at least 5 years for AF and 7 years for MI after the episode and before the study either ended or the patient died.

Single AF incidents appear more influenced by lifestyle factors and age, with only two unique genetic regions identified. In contrast, patients with recurrent AF are typically younger at their first event and exhibit a stronger genetic basis, as evidenced by 18 unique genetic regions linked to recurrent AF. Among these, two regions are near the *NAV2* and *SCN10A* genes, previously hypothesized, but unconfirmed (37, 38) (N=42,585 East Asian population, N=660 German population), to affect recurrent AF. The region with the lowest p-values and also the largest amount of significant SNPs was found near the *PITX2* gene for both the single and the recurrent AF groups. Though *PITX2* is the most known gene associated with recurrent AF (1, 7, 10) (N=295 Turkish population, N=195 Caucasian population, N=991 German and American population), our results do not indicate such an effect. This observation extends to six other genes (*SOX5, CAV1, EPHX2, ITGA9, SLC8A1*, and *TBX5*) (8, 10, 37) (N=660 German population, N=295 Turkish population, N=42,585 East Asian population, N=486 Caucasian population), where variants near the *SOX5, CAV1*, and *TBX5* genes are identified in both the single and recurrent AF groups, and no variants near the *EPHX2, ITGA9*, and *SLC8A1* genes are identified in any of the groups. PheWAS analysis of these unique single and recurrent AF SNPs further highlight distinct susceptibilities: SNPs associated with single AF correlate with 44 phenocodes across various categories, whereas recurrent AF SNPs are linked to 91 phenocodes, predominantly in the circulatory system category. Network analysis of gene sets revealed that 16 of the 18 genes associated with recurrent AF are connected through 82 shared, highly co-expressed neighboring genes. These recurrent AF genes, along with their neighboring genes, are involved in complex processes related to heart rate regulation and cardiac muscle cell action potential. In contrast, the two genes associated with single AF events are linked to heart and cardiac muscle processes but do not share highly co-expressed genes.

We also find distinct clinical and genetic differences between patients with single and recurrent MI. Unlike AF, recurrent MI is more associated with older age at the first MI event, lifestyle factors, and age-related issues. This is despite the fact that the single MI group is adjusted for early death due to MI and/or related comorbidities, and thus, this should not affect the results. The genetic predisposition seems stronger in single MI cases, with a total of 24 uniquely associated regions. In contrast, recurrent MI is only associated with two regions that did not share the association with single MI cases. Of the 24 genetic regions uniquely identified for single MI, 14 are previously known for MI or other CVD-related traits. The remaining 10 are novel for MI and have previously not been reported for other CVD-related traits. While most of these novel regions consist of single SNPs primarily identified in the HUNT population, their nearest genes code for functions similar to known MI regions. Looking into the allele frequencies in each population (Supplementary File 4), we see that these variants are rather common in HUNT and in the general European population [based on reports from gnomAD (39)], while they are rare variants in the UKBB population and thereby not included in the meta-analysis. Further studies are needed to investigate if these variants show similar effects in other European and non-European populations.

PheWAS analysis reinforces the genetic distinction between single and recurrent MI groups. The single MI group’s SNPs are linked to 128 phenotypes, predominantly in the circulatory system category, with additional associations in the endocrine/metabolic category and notable links to neurodegenerative disorders. In contrast, the SNPs related to recurrent MI correlate with 16 phenocodes, involving both circulatory and endocrine/metabolic categories, but the associations are not as pronounced as those in the single MI group. Our network analysis reveals distinct gene interactions for each group. Of the 24 genes uniquely associated with single MI, 21 share connections with 110 genes in the co-expression network. However, the two genes associated with recurrent MI have seven highly co-expressed genes. This indicates more extensive genetic interconnections in single MI cases. Interestingly, the shared neighboring genes for single MI, and those common to both single and recurrent MI, show functional enrichment in several biological processes. In contrast, the shared neighboring genes for recurrent MI do not show significant functional enrichment. This disparity suggests distinct biological pathways involved in single vs. recurrent MI events. Moreover, only one function is common between the shared genes for both single and recurrent MI, suggesting unique biological mechanisms specific to single MI events.

The results suggest a greater genetic influence in AF compared to MI, but several factors could affect this perception. Clinically, it is expected that more genes would increase the risk of recurrent AF, a pattern observed in this study. In contrast, the findings for MI are the opposite, potentially influenced by their higher age: MI becomes less common in younger individuals, and in older populations, comorbidities often overshadow genetic risk factors. There could also be physiological reasons behind these observations. For instance, MI in younger individuals might more frequently result from genetic factors related to platelet aggregation or atherosclerosis, conditions that are generally more responsive to treatment. In older individuals, MI might be more associated with broader age-related issues, reducing the relative impact of genetics. The reporting of these conditions could also influence the results, with AF potentially being under-reported compared to MI, which itself is possibly over-reported. This discrepancy could explain why phenocodes related to neurodegenerative disorders and cerebral ischemia emerge as significant only in single MI cases in the PheWAS results. These findings might be influenced by diagnostic practices where MI is often recorded as a cause of death, even when other diseases are the actual cause, or in cases where individuals die before a recurrent event occurs. The latter has been adjusted for by excluding individuals with single events with less than 7 years between the event and the censoring date, being either death or the end of the study. Still, even with this adjustment, some single MI event cases might be censored out before a second event, possibly influencing the case groups and thereby the results.

The observed differences in clinical characteristics for both diseases might be even more pronounced if the HUNT and UKBB studies had used similar questionnaire formats. In the HUNT study, participants involved in both HUNT2 and HUNT3 provided cholesterol and blood pressure measurements taken 11 years apart. We opted to use the measurements recorded closest to the first AF or MI event, as we believe these offer the most relevant information. However, for the UKBB participants, most were measured only once, eliminating the option to choose measurements closest to the event. This discrepancy in data collection methodology could explain why significant differences in cholesterol and blood pressure measurements are observed between single and recurrent event groups in the HUNT population but not in the UKBB group. The lack of longitudinal data in the UKBB may obscure potential differences that are more apparent in the HUNT study due to its repeated measurements.

In any GWAS study, larger sample sizes, diverse ancestry representation, and result replication are crucial. The HUNT study, comprised solely of individuals of European ancestry, led us to select only European ancestry participants from the UKBB for consistency. However, for global applicability, conducting similar analyses across all ancestries is essential. Regarding sample sizes, while the combined participant pool of HUNT and UKBB exceeds 500,000, the specific filtering and subgrouping in our study result in some case/control groups having fewer than 2,000 individuals. This reduced size may limit our ability to achieve robust significant findings. The small sample sizes might explain the prevalence of significant singleton SNP hits (one SNP per region) found only in the HUNT study, with the same SNPs with too rare allele frequencies in the UKBB population. Increasing sample sizes or including an additional population would likely enhance the reliability of these findings, particularly since these variants are reported as common in the general European population. While increasing the sample sizes could verify/dispute or even identify additional regions, some genetic differences observed between recurrent and single cases might diminish. Although many spikes in the Manhattan plots (Figures 1 and 2) indicate clear differences between single and recurrent cases, certain regions almost reach significance for the opposite group, such as the hit on chromosome 8 for recurrent AF. This suggests that some observed genetic distinctions might be less pronounced with a more substantial and diverse sample.

While our meta-analysis did not yield any GWAS significant results when directly comparing recurrent to single AF or MI cases, the data still indicate genetic differences between these groups. Notably, some genetic regions were identified in both single and recurrent groups (termed “common”), while a substantial number were unique to each group. This could suggest that the common regions may play a role in the general susceptibility to AF and MI, whereas the unique regions could confer specific genetic risks for the disease’s form (as a single or recurrent events). Further studies are needed to test the hypotheses generated from this study: Investigate the effect of the novel SNPs and genes identified and their involvement in either single or recurrent AF/MI in particular. While many of the identified genes and their related function might not have a direct confirmed effect on AF/MI, we do see similar functions in the novel genes as in the known AF/MI genes, thus showing the potential for identification and generating an understanding of new genetic functions of the diseases.

There has been considerable research aimed at uncovering genetic causes for AF and MI. Our findings underscore the importance of genetic studies focused on disease subgroups, as conducted here. Both AF and MI are widespread diseases with varied impacts on individuals’ lives. The progression and outcomes of these diseases are not uniform across all patients. By examining subgroups within these diseases, we could gain new insights into their mechanisms, potentially leading to more effective prevention and treatment strategies tailored to different patient profiles.

A limitation of using the LD Score Regression software to calculate the observed scale heritability is that it is not well suited for mixed models. The GWAS analysis was performed using SAIGE, where we assume a logistic mixed model to account for imbalanced case–control ratios and relatedness. Hence, the precision of the heritability calculated here is limited due to this fact.

## Data Availability

The original contributions presented in the study are included in the article/**Supplementary Material**, further inquiries can be directed to the corresponding author.

## Supplementary material

Supplementary File 2

Excel file containing all phenocodes found to be significantly associated with the set of SNPs associated with both single and recurrent AF/MI or uniquely for one of them.

Supplementary File 3

Excel file containing genes shared between the GWAS associated genes (target genes) in the network analysis for genes associated with both single and recurrent AF/MI or uniquely for one of them.

Supplementary File 4

Excel file containing allele frequency of SNPs identified in only one of the populations (listed as for instance −? in Tables 2, 3). The columns named ‘AF HUNT’, ‘AF UKBB’ and ‘AF European’ shows the allele frequency in the HUNT population, the UKBB population and as reported in gnomAD (39)(https://gnomad.broadinstitute.org/), respectively. The ‘HUNT/UKBB’ columns shows if the SNP is identified in HUNT or UKBB, and the ‘imputation info’ columns shows the imputation info given in the genotyped UKBB files.

Supplementary File 5

Excel file containing the data from Tables 2, 3.
